# Devitrification reduces beam-induced movement in cryo-EM

**DOI:** 10.1107/S2052252520016243

**Published:** 2021-03-01

**Authors:** Jan-Philip Wieferig, Deryck J. Mills, Werner Kühlbrandt

**Affiliations:** a Department of Structural Biology, Max Planck Institute of Biophysics, Frankfurt, Germany

**Keywords:** cryo-EM, devitrification, beam-induced movement

## Abstract

As cryo-EM approaches the physical resolution limits imposed by electron optics and radiation damage, it becomes increasingly urgent to address the issues that impede high-resolution structure determination of biological specimens. One of the persistent problems has been beam-induced movement, which occurs when the specimen is irradiated with high-energy electrons. Beam-induced movement results in image blurring and loss of high-resolution information. It is particularly severe for biological samples in unsupported thin films of vitreous water. By controlled devitrification of conventionally plunge-frozen samples, the suspended film of vitrified water was converted into cubic ice, a polycrystalline, mechanically stable solid. It is shown that compared with vitrified samples, devitrification reduces beam-induced movement in the first 5 e Å^−2^ of an exposure by a factor of ∼4, substantially enhancing the contribution of the initial, minimally damaged frames to a structure. A 3D apoferritin map reconstructed from the first frames of 20 000 particle images of devitrified samples resolved undamaged side chains. Devitrification of frozen-hydrated specimens helps to overcome beam-induced specimen motion in single-particle cryo-EM, as a further step towards realizing the full potential of cryo-EM for high-resolution structure determination.

## Abstract 2

Controlled devitrification of vitreous samples for high-resolution single-particle cryo-EM reduces beam-induced motion in the critical early frames of a movie stack by a factor of approximately four.

## 1. Introduction   

The new direct electron detectors and image-processing programs have resulted in a ‘resolution revolution’ in electron cryo-microscopy (cryo-EM; Kühlbrandt, 2014 ▸). Since then, the limits of the method have shifted towards smaller particles (Merk *et al.*, 2016 ▸; Khoshouei *et al.*, 2017 ▸) and higher resolution (Yip *et al.*, 2020 ▸; Nakane *et al.*, 2020 ▸). The enhanced detective quantum efficiency of direct detectors greatly improves the signal-to-noise ratio, and their high readout speed allows image acquisition as stacks of dose-fractionated movie frames (McMullan *et al.*, 2014 ▸). Evaluation of movie stacks identified beam-induced specimen movement as a main limiting factor in cryo-EM (Henderson *et al.*, 2011 ▸; Brilot *et al.*, 2012 ▸; Campbell *et al.*, 2012 ▸).

Beam-induced movement is most likely caused by pre-existing mechanical stress frozen into the sample that is released upon electron irradiation (Glaeser, 2016 ▸; Vinothkumar & Henderson, 2016 ▸; Thorne, 2020 ▸; Naydenova *et al.*, 2020 ▸). By comparison, specimen charging (Glaeser, 2016 ▸) appears to play only a minor role (Russo & Henderson, 2018*a* ▸,*b* ▸). Some motion is thought to be caused by the vitrified water itself (Russo & Passmore, 2014 ▸), but the Brownian-type movement transmitted to the protein by the surrounding water molecules in the highly viscous liquid film is not expected to contribute much to beam-induced motion (McMullan *et al.*, 2015 ▸).

Cryo-EM specimens are normally prepared by applying a small drop of an aqueous solution of a macromolecule to a specimen support, most often a copper grid coated with a layer of holey carbon film. Excess liquid is blotted off and the grid is plunged into a bath of liquid ethane, which results in rapid vitrification of the thin aqueous layer in the holes containing the particles (Dubochet *et al.*, 1988 ▸). Rapid freezing generates and traps mechanical stress in the specimen. Moreover, the copper grid contracts while water expands to low-density vitreous water upon freezing (Glaeser, 2016 ▸). The amorphous carbon coating contracts less than the copper grid bars, resulting in cryo-crinkling of the carbon film (Booy & Pawley, 1993 ▸) and buckling of the vitrified water layer (Naydenova *et al.*, 2020 ▸).

Two phases of beam-induced movement can be distinguished during the acquisition of a movie stack (Russo & Passmore, 2014 ▸). The first phase includes the initial 5–10 e Å^−2^. When the first electrons hit, the frozen-in stress is released, resulting in a burst-like beam-induced specimen motion observed in the initial phase. The moderate, continuous beam-induced motion during the second phase may signify the accumulation of new stress in the specimen as a result of radiation damage (Glaeser *et al.*, 2011 ▸; Glaeser, 2016 ▸) or pseudo-diffusive motion owing to the changed viscosity of vitreous water during irradiation (Naydenova *et al.*, 2020 ▸). It has been shown that stable, all-gold specimen supports with standard hole sizes (1.2–2 µm) reduce beam-induced specimen motion by 40% in the first phase and by 80% in the second phase (Russo & Passmore, 2014 ▸). Reduced beam-induced specimen motion has also been reported for specimens vitrified in liquid ethane at −110°C (Shi *et al.*, 2019 ▸) rather than the usual temperature of a few degrees above the melting point at −183°C.

With the advent of direct detectors and movie stacks, beam-induced specimen motion can in principle be compensated by aligning movie frames and correcting the movements of individual particles or of patches containing ensembles of particles moving together (Ripstein & Rubinstein, 2016 ▸). However, beam-induced motion in the first few frames (at a fluence of ∼3–4 e Å^−2^) can be so severe that they are simply discarded (Liao *et al.*, 2013 ▸; Allegretti *et al.*, 2014 ▸; Vinothkumar *et al.*, 2014 ▸; Merk *et al.*, 2016 ▸). A better option is to downweight the initial frames during movie refinement, as in the image-processing program *RELION*-2 (Scheres, 2014 ▸), and for Bayesian polishing in *RELION*-3 (Zivanov *et al.*, 2019 ▸). Electron diffraction patterns of purple membrane (2D crystals of bacteriorhodopsin) have indicated that up to 99% of the 3 Å signal is lost to radiation damage by the first five 300 kV electrons per Å^2^ (Vinothkumar & Henderson, 2016 ▸; Stark *et al.*, 1996 ▸). However, more recent measurements with both single particles (Grant & Grigorieff, 2015 ▸) and 2D crystals (Peet *et al.*, 2019 ▸) suggest that damage in the early frames is less severe (Naydenova *et al.*, 2020 ▸). In any case, the initial frames, which suffer most from beam-induced specimen motion, are particularly precious for structure determination because they contain most of the high-resolution information. To realize the full potential of cryo-EM, it is thus desirable to reduce beam-induced movement to a minimum (Henderson & McMullan, 2013 ▸). In this respect, a breakthrough has recently been achieved in the design of novel all-gold specimen supports with very small holes (200–300 nm diameter), which limit the total movement to <1 Å (Naydenova *et al.*, 2020 ▸).

Long before the introduction of direct electron detectors, a set of experiments revealed that the high-resolution signals of two biological specimens, Tobacco mosaic virus and thin crystals of catalase, improved substantially when they were surrounded by cubic ice instead of vitreous water (Cyrklaff & Kühlbrandt, 1994 ▸). Cubic ice is a metastable polycrystalline solid with a small average crystal size of a few hundred Å, whereas vitreous water is an amorphous solid that can be thought of as an extremely viscous liquid even at very low temperatures (Smith *et al.*, 2000 ▸). Cubic ice forms when vitreous water is heated above its glass-transition temperature. The transition time depends on the temperature and decreases from around 1 h at −137°C to less than a second at −120°C (Dubochet *et al.*, 1988 ▸; Smith *et al.*, 2000 ▸). Cubic ice converts gradually into hexagonal ice, which is thermodynamically stable but has a higher nucleation barrier. This conversion is slow and exceeds laboratory time scales at temperatures below −33°C (Bartels-Rausch *et al.*, 2012 ▸).

Slow freezing can damage proteins, either as a result of dehydration owing to a local increase in solute concentration or owing to a change in ionic conditions, for example pH. Surface effects at the ice–water interface and shear forces during ice-crystal formation might also contribute to sample denaturation (Cao *et al.*, 2003 ▸; Franks, 1985 ▸). This is unlikely to happen during the transformation of vitreous water surrounding the sample to cubic ice by devitrification at low temperature. Indeed, early experiments have shown that controlled devitrification does not damage biological samples (Cyrklaff & Kühlbrandt, 1994 ▸). Electron diffraction and imaging of specimens embedded in cubic ice indicated that the nanocrystalline surroundings offer a mechanically more rigid support and increase the success rate of acquiring high-resolution images.

In this paper, we examine the potential of devitrification for high-resolution single-particle cryo-EM, taking advantage of the speed and sensitivity of direct electron detectors that were not available at the time of the original experiments in 1994. We find that devitrification results in a substantial reduction of beam-induced movement and preserves high-resolution detail in the first few frames of a movie stack.

## 2. Methods   

### 2.1. Specimen preparation and controlled devitrification   

Quantifoil R2/2 400 mesh copper grids were pre-treated by washing in trichloromethane for >2 h. The grids were glow-discharged twice before 3 µl of sample solution was applied and plunge-frozen using a Vitrobot Mark IV. Initial experiments were performed with alcohol oxidase as a stable, soluble test sample that was readily available in our laboratory, at a concentration of ∼0.7 mg ml^−1^ in 20 m*M* sodium phosphate buffer. Alcohol oxidase has a molecular mass of 600 kDa and dimensions of 130 × 130 × 105 Å (Vonck *et al.*, 2016 ▸). Subsequent experiments were carried out with horse spleen apoferritin from Sigma–Aldrich (catalogue No. A3641), which does not suffer from preferential orientation and is now widely accepted as the standard cryo-EM test specimen. Apoferritin has a molecular mass of 481.2 kDa and is roughly spherical, with an outer diameter of 120–130 Å and a ∼20 Å protein shell surrounding a ∼80 Å internal aqueous space. Grids were prepared by diluting a 54 mg ml^−1^ stock 1:130 with 135 m*M* sodium chloride in distilled water (∼0.4 mg ml^−1^). Vitrified grids were clipped in Krios autoloader cartridges. For devitrification, clipped grids were placed into the milled wells of an aluminium plate of a home-built grid heater (Supplementary Fig. S1) cooled to around −190°C with liquid nitrogen. The top plate was gradually warmed to −120°C. After 10 min at this temperature, the plate was cooled to −190°C (Supplementary Fig. S2) and the clipped grids were transferred to a storage box under liquid nitrogen. In other devitrification experiments, grids were placed directly onto the metal plate of the grid heater for 5–7 s at a temperature of −100 to −110°C before manual immersion in liquid nitrogen. Grids were handled and mounted in a humidity-controlled atmosphere (∼20% relative humidity at 21°C). Both devitrification procedures gave similar results.

### 2.2. Imaging   

Micrographs were collected on a Titan Krios electron microscope equipped with a Gatan K2 camera with energy filter in counting mode at a pixel size of 1.077 Å. 40-frame movies were collected with a total exposure time of 8 s using the *EPU* automatic acquisition software at a transmitted fluence per frame of 1.1–1.3 e Å^−2^. Defocus was in the range 0.6–2.5 µm. The energy filter was set to a slit width of 20 eV. Four micrographs were acquired per foil hole.

### 2.3. Image processing   

Micrographs were pre-processed in *RELION*-3 (Zivanov *et al.*, 2018 ▸) with *MotionCor*2 (Zheng *et al.*, 2017 ▸) using 5 × 5 patches and *Gctf* (Zhang, 2016 ▸). Particles were automatically picked using templates generated from 2D classes of manually picked particles. Carbon edges and ice contamination were identified by 3D classification and removed. The best 3D classes were further refined in the *RELION* 3D autorefinement procedure. Per-particle CTF refinement and beam-tilt estimation was performed with *RELION* before applying a frequency-dependent dose-weighting scheme in the Bayesian polishing procedure. Polished particles were again submitted to per-particle CTF refinement and 3D autorefinement and, in the case of apoferritin, octahedral symmetry was applied. Subsequently, the maps were masked and sharpened using *B* factors calculated in the *RELION* post-processing procedure according to Rosenthal & Henderson (2003 ▸). Average particle drifts per frame were calculated from the change in particle position between frames, as determined by Bayesian polishing (Supplementary Fig. S3).

### 2.4. Single-frame reconstructions   

Raw movies were motion-corrected using *MotionCor*2 in *RELION*-3 as before, but the corrected movie stacks were written out. Frames 1–20 were extracted individually from the corrected movie stacks. From these single frames, 20 000 randomly chosen, fully processed particles were extracted. For each frame two half-maps were reconstructed, applying previously assigned orientation and octahedral symmetry in *relion_reconstruct*. The half-maps were then masked as for the full data set, and sharpened by the *RELION* post-processing procedure. Maps were drawn with *ChimeraX* (Goddard *et al.*, 2018 ▸) at the default contour level where 1% of voxels are above the threshold, and the apoferritin map was fitted with the 1.5 Å resolution X-ray structure of horse apoferritin (PDB entry 2w0o; de Val *et al.*, 2012 ▸).

### 2.5. Radial power spectra   

For calculating radial power spectra, the same number of single apoferritin particles and empty background regions of equal dimensions were manually picked from each motion-corrected micrograph in *RELION*. 1900 particles and backgrounds were picked from 27 micrographs in data set ‘ApoF devitrified 1’, 2600 particles and backgrounds were picked from 41 micrographs in data set ‘ApoF devitrified 2’, 1800 particles and backgrounds were picked from 32 micrographs in data set ‘ApoF vitreous 1’ and 1600 particles and backgrounds were picked from 38 micrographs in data set ‘ApoF vitreous 2’.

Particle and background images were then extracted and a tight, soft spherical mask was applied to prevent spikes in the Fourier transform and to exclude as much of the background as possible in the particle images. Fourier transforms of particles and background areas were calculated and the intensities per micrograph were averaged. Finally, intensities of Fourier rings were averaged for the background and particle images to obtain radial power averages. Particle and background stacks were processed using the MRC processing software programs *LABEL* and *FFTRANS* (Crowther *et al.*, 1996 ▸) and the spherical mask was applied with *EMAN*2 (Tang *et al.*, 2007 ▸). The Fourier ring averages were calculated as in Chen *et al.* (2013 ▸) using a program kindly provided by Richard Henderson.

## 3. Results   

### 3.1. Controlled devitrification   

Images of horse apoferritin or alcohol oxidase on plunge-frozen carbon-coated copper grids were devitrified on a nitrogen-cooled metal plate (Supplementary Fig. S1) by raising its temperature to roughly 20–30°C above the glass-transition point near −137°C. The grids were then transferred into the autoloader of a Titan Krios electron microscope. Control grids of vitrified but otherwise identical samples were loaded and examined in separate sessions. Grid squares screened in the electron microscope for successful devitrification fell into one of three categories.

**Figure 1:**
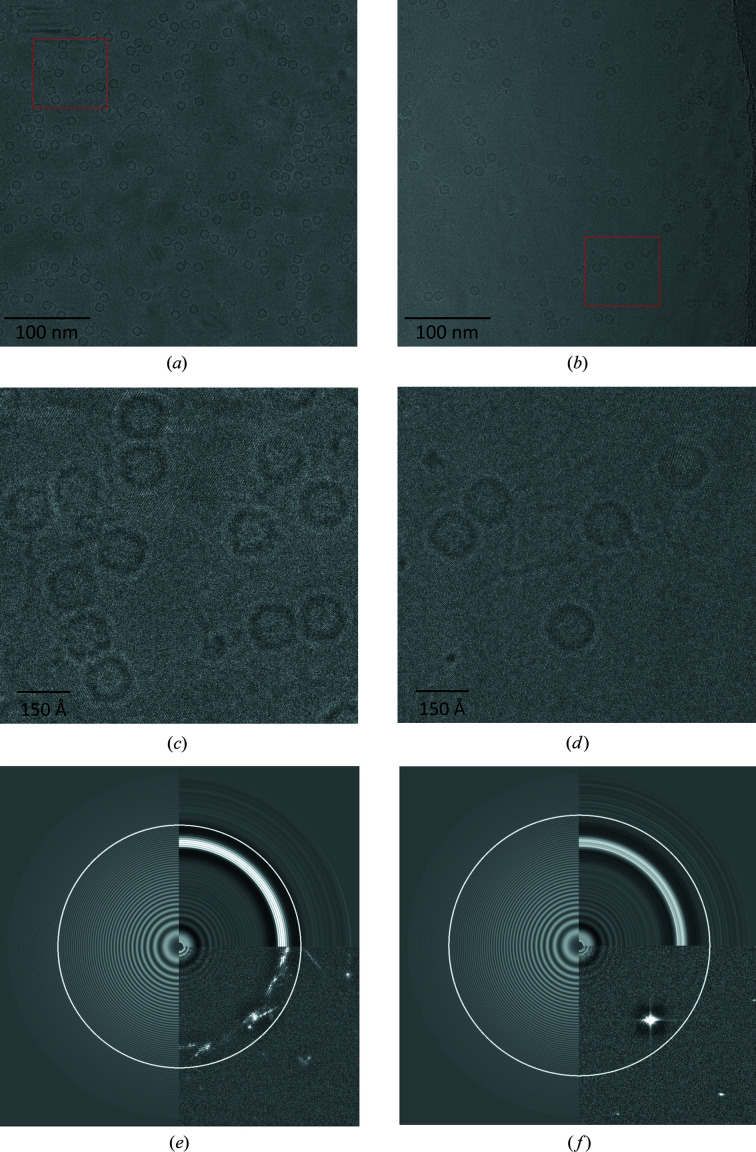
Motion-corrected micrographs of apoferritin (120–130 Å outer diameter) in cubic ice at 1.077 Å pixel size and a defocus of 1.93 µm (*a*) and 1.96 µm (*b*). Red boxes show the areas enlarged in (*c*) and (*d*). The ice lattice is visible by eye in both micrographs. (*a*) shows numerous small crystallites with different orientations, whereas in (*b*) the crystal lattice is uniform. This is also evident from the respective Fourier transforms (*e*, *f*). The lower right-hand quadrant in (*e*) has multiple reflections, whereas in (*f*) it shows only one single spot at 3.68 Å resolution. The highest resolution to which the CTF could be fitted is indicated by the thin white circles in (*e*) (3.19 Å) and (*f*) (2.96 Å). The top right quadrant shows the rotational average of the Fourier transform and the left half shows the fitted CTF.

**Figure 2:**
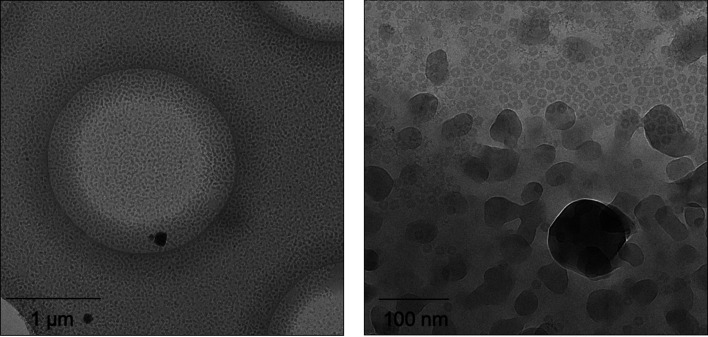
Ice contamination upon devitrification for 5–6 s at −105°C. The image on the left shows a Quantifoil R2/2 foil hole. The image on the right shows another foil hole at higher magnification, with alcohol oxidase against a background of contaminating ice crystals. Fourier transforms of manually picked particles revealed that the aqueous film around the particles in this image had not devitrified.

(i) The vitreous water film in a number of adjacent grid squares had devitrified. Devitrified areas were distinguished from vitreous squares by tilting the stage at ∼6400× magnification, which caused the ice reflections to move in the direction perpendicular to the tilt axis. If the ice layer was very thin and the reflections were weak, the holes were checked at a magnification of 130 000× as used for image acquisition. At this magnification the cubic ice lattice was easily visible by eye and crystallinity was evident in the image transform (Fig. 1 ▸).
(ii) Other squares on the same grid appeared to be covered with contaminating atmospheric ice crystals (Fig. 2 ▸). Depending on the crystal size, these grid squares looked rough rather than smooth at lower magnification (∼6400×). Frequently, ice crystals would form central patches that covered large parts of the grid square.
(iii) Some grids appeared to be unchanged compared with grids that had not been placed onto the heater plate and none of the grid squares had devitrified.

### 3.2. Single particles in cubic ice   

Data sets for horse spleen apoferritin in cubic ice were collected from two devitrified grids in consecutive sessions. From the first grid, 239 micrographs of devitrified foil holes in three grid squares were acquired. From the second grid, 698 micrographs of devitrified holes in ten grid squares were acquired. Foil holes close to grid bars were omitted, as the water layer tended to be too thick. On successfully devitrified grid squares, all foil holes that were not empty contained crystalline ice. At a defocus above ∼1.2 µm, particles were easily visible against the ice lattice in the background (Fig. 1 ▸). If necessary, the crystal lattice image was removed by 4 Å low-pass filtering for better visualization of the apoferritin particles.

**Figure 3:**
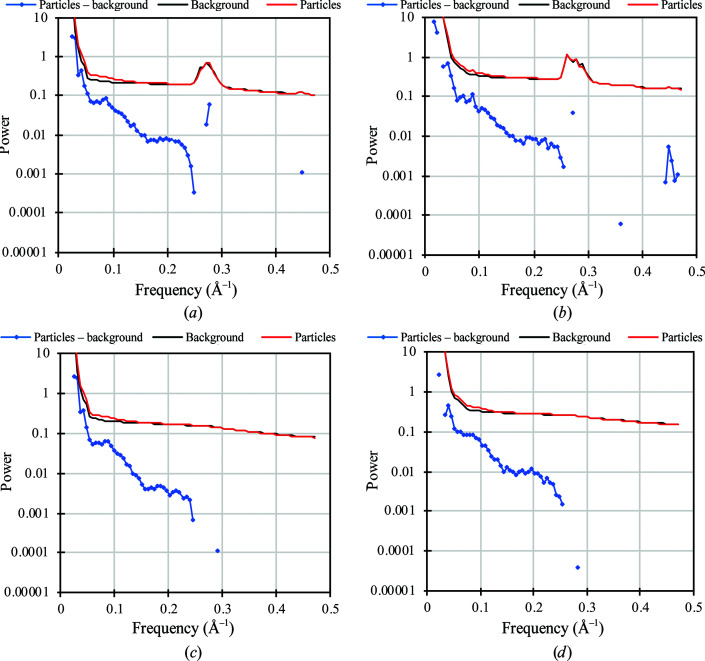
Radial power spectra of apoferritin in devitrified (*a*, *b*) and vitreous (*c*, *d*) water. Average radial signal intensities were calculated from 1600–2600 particles and the same number of background regions of equal size. The blue line shows the difference between the power spectra of particles (red) and background (black) for regions of the transform where the particle signal is above background. For particles in cubic ice this was the case to 0.250 Å^−1^ (*a*) or 0.255 Å^−1^ (*b*), and for particles in vitreous water to 0.244 Å^−1^ (*c*) or 0.255 Å^−1^ (*d*). In the regions beyond, background was stronger except for sporadic higher frequencies. Particle and background intensities at low spatial frequency extend to 1.9–4.6 × 10^5^.

Radial power spectra of tightly boxed apoferritin particles and empty background regions indicated a peak in the 0.250–0.300 Å^−1^ resolution range centered around the main diffraction peak of cubic ice at 0.272 Å^−1^ (Dowell & Rinfret, 1960 ▸). The ice peak therefore indicated that the vitreous water had been converted to cubic ice. As expected, this peak was absent in the power spectra of corresponding regions on vitreous grids (Fig. 3 ▸). Since the ice peak was visible in transforms of tightly boxed particle images, the water layer immediately below or above the apoferritin had also devitrified. We assume that this was also the case for water in the ∼80 Å internal space of apoferritin. The difference between average particle signal intensity and average background intensity was plotted in the radial power spectra for all frequencies where this difference was positive. The Fourier intensities of the particles were above background to ∼0.25 Å^−1^ for both vitrified and devitrified samples.

484 micrographs of alcohol oxidase in cubic ice were acquired from one grid. Additional reference images of alcohol oxidase in vitreous buffer were collected from one other grid. The results were similar to those for apoferritin above. Owing to the lower *D*4 symmetry of alcohol oxidase and its tendency to arrange in arrays with strong preferential orientation of individual particles, we found that alcohol oxidase was less suitable than apo­ferritin as a test specimen to study the effect of devitrification on protein structure at high resolution.

### 3.3. Image processing   

**Figure 4:**
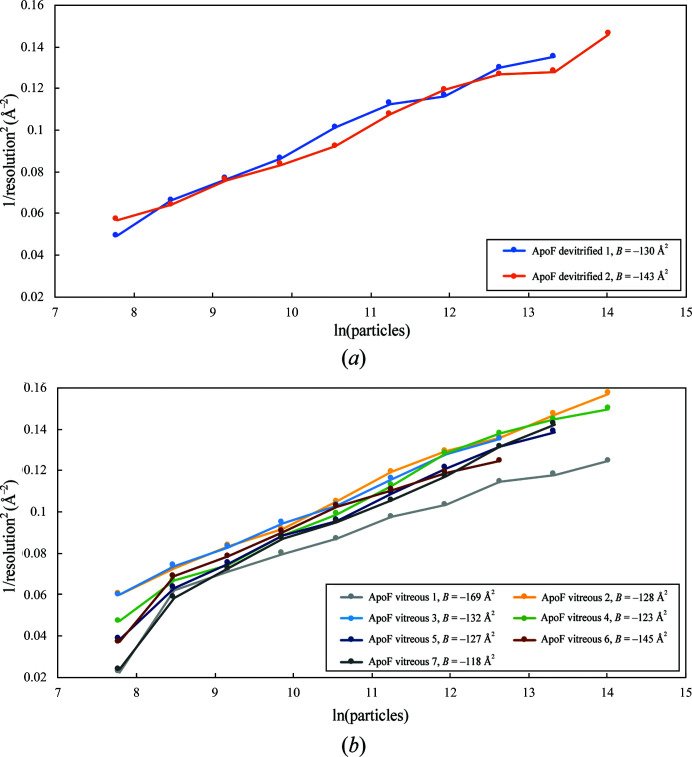
Rosenthal plots of the inverse squared resolution versus the natural logarithm of the number of asymmetric units of horse spleen apoferritin (ApoF; 24-fold symmetry, octahedral) in cubic ice (*a*) and vitreous water (*b*). *B* factors were calculated from the slope, excluding the first data point of all data sets with vitreous water. FSC curves for ApoF vitreous 2 and ApoF devitrified 2 are shown in Supplementary Fig. S4.

In total, images from 11 Quantifoil R2/2 copper grids were processed in *RELION*. One was of alcohol oxidase in cubic ice, another was of alcohol oxidase in vitrified buffer, two were of apoferritin in cubic ice and seven were of apoferritin in vitreous water. The resolutions achieved with specimens of apoferritin in devitrified buffer were 2.7 and 2.62 Å, with 25 600 and 51 200 particles, respectively [Fig. 4 ▸(*a*)]. The resolution of apoferritin maps in vitreous water ranged from 2.83 to 2.52 Å, with up to 51 200 particles, depending on the sample [Fig. 4 ▸(*b*)]. FSC curves for vitreous and devitrified apoferritin specimens are shown in Supplementary Fig. S4. The preferred orientation of alcohol oxidase particles on the grid (Fig. 2 ▸) limited the resolution to 3.45 and ∼4 Å for devitrified and vitreous specimens, respectively (not shown).

### 3.4. Comparison of particle movement in vitreous and devitrified water   

**Figure 5:**
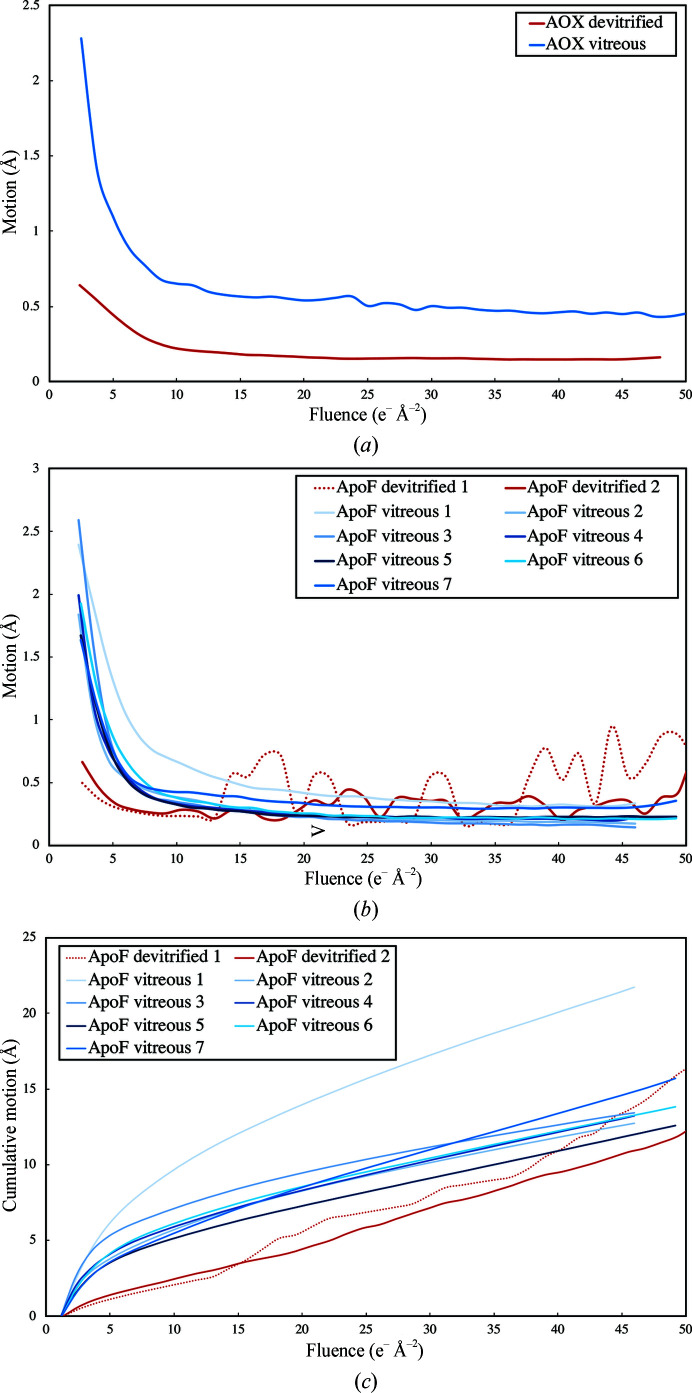
Averaged per-frame motion of all particles used in the last refinement round of (*a*) alcohol oxidase (AOX) and apoferritin (ApoF) (*b*, *c*). The particle drift is calculated from the particle trajectories determined by Bayesian polishing in *RELION*-3 (Suppplementary Fig. S3). A systematic reduction of particle drift is evident for both apoferritin and alcohol oxidase in cubic ice (red) compared with vitreous water (blue) in the most important early frames.

**Figure 6:**
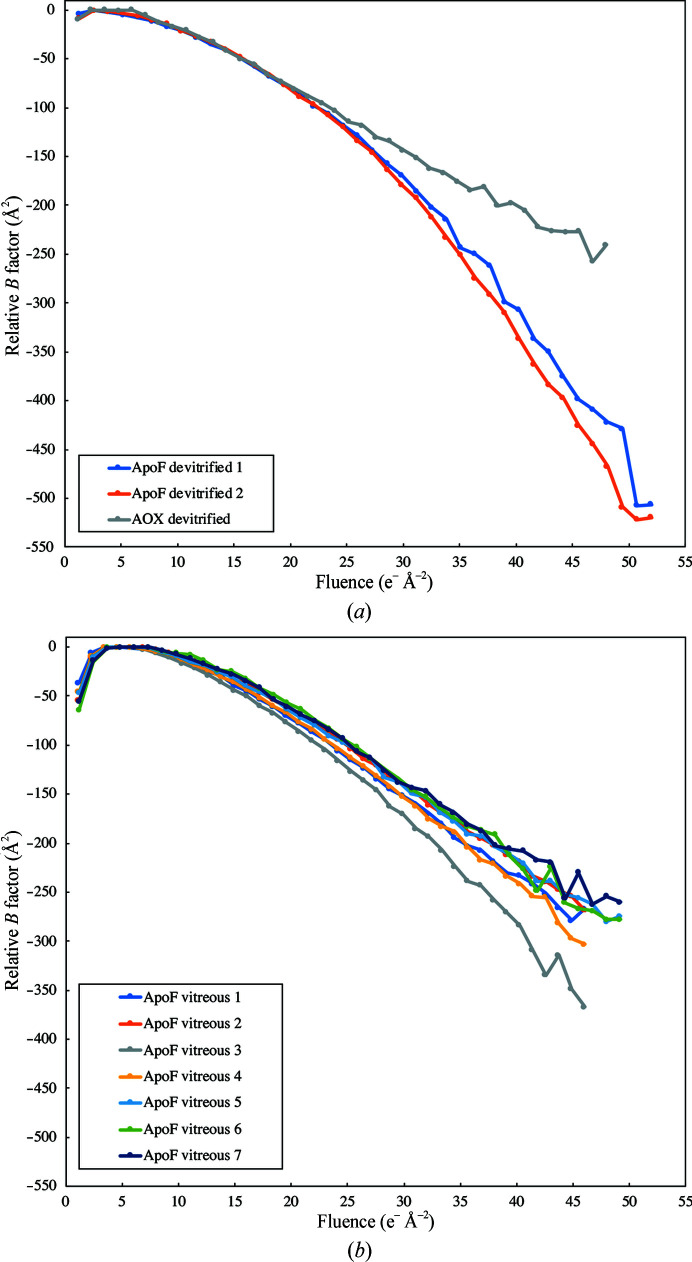
Relative Bayesian polishing *B* factors determined in *RELION*-3 for apoferritin (ApoF) or alcohol oxidase (AOX) in cubic ice (*a*) or for apoferritin in vitreous water (*b*). The per-frame *B* factor of the first, minimally damaged frame is better by 28–60 Å^2^ for devitrified than for vitreous samples.

Whole-frame alignment of dose-fractionated movies revealed a substantial reduction in beam-induced movement for devitrified specimens. Average particle trajectories of alcohol oxidase [Fig. 5 ▸(*a*)] indicated an initial drift of 0.64 Å per frame (∼1.2 e Å^−2^) in cubic ice and 2.28 Å in vitreous water, which subsided to 0.46 Å (devitrified) and 1.01 Å (vitrified) per frame after ∼5 e Å^−2^. Average particle trajectories of apoferritin [Fig. 5 ▸(*b*)] indicated an initial drift of 0.50 Å (ApoF devitrified 1) and 0.66 Å (ApoF devitrfied 2) per frame, which decreased to 0.30 and 0.33 Å, respectively, after ∼5 e Å^−2^. On the devitrified apoferritin grids, movement in the second phase was more erratic. In the vitreous apoferritin sample (ApoF vitreous 1–7) the initial drift ranged from 1.64 to 2.59 Å per frame and decreased to 0.69–1.4 Å after ∼5 e Å^−2^. Devitrification thus reduced the drift by a factor of ∼4 in the initial frames and by a factor of ∼2 in subsequent frames. The relative per-frame *B* factors of the early frames were correspondingly better for devitrified specimens (Fig. 6 ▸).

The damage rate after the first 10 e Å^−2^, indicated by the slope of the *B*-factor curves in Fig. 6 ▸, is approximately 4.6 Å^2^ per e Å^−2^. This is in good agreement with previous radiation-damage measurements on single particles (Grant & Grigorieff, 2015 ▸) and 2D crystals (Peet *et al.*, 2019 ▸).

### 3.5. Single-frame reconstructions   

**Figure 7:**
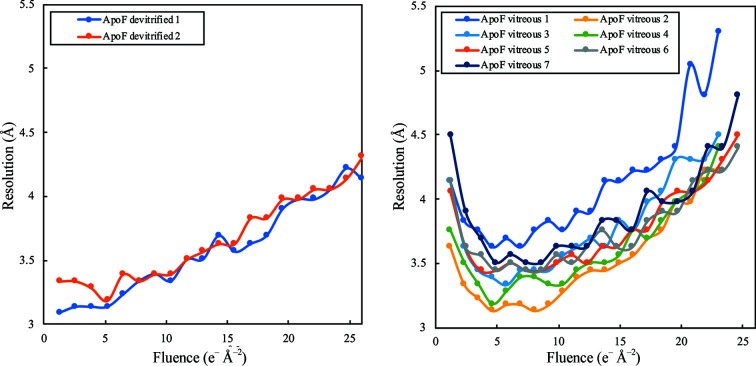
20 000 particles were randomly chosen from the final 3D refinement of the full data sets and re-extracted from individual frames for 3D reconstruction. In data sets acquired from apoferritin (ApoF) in cubic ice (left) the resolution of the reconstructions using only the first frame (1.3 e Å^−2^) were better than those of vitrified specimens (right). The overall trend in the reconstructions from apoferritin in cubic ice was a decrease in resolution with increasing fluence, as expected owing to accumulating radiation damage. With apoferritin in vitreous water, the highest resolution single-frame reconstruction was obtained with the fourth frame (4.6 e Å^−2^), at which point the initial burst of beam-induced movement had subsided. For vitrified samples, the resolution of the first-frame reconstruction (1.15–1.23 e Å^−2^) was consistently lower than for any of the first ten frames owing to the large initial beam-induced movement.

Single-frame reconstructions were calculated from 20 000 randomly selected, fully processed particles re-extracted from individual frames. Pre-assigned orientations were used to reconstruct two half-maps and octahedral symmetry was applied. The reconstructions were then masked and sharpened in *RELION*. The resolution of individual frame reconstructions is plotted in Fig. 7 ▸.

The map reconstructed from the first frames (fluence of 1.3 e Å^−2^) of each movie stack in the ApoF devitrified 1 data set achieved a resolution of 3.1 Å, the highest of all single-frame reconstructions. In the ApoF devitrified 2 data set frame 4 was the best, but both were very much better than any of the seven vitreous samples. The resolution of first-frame (fluence of 1.15–1.23 e Å^−2^) reconstructions of apoferritin in vitreous water ranged from 3.6 to 4.5 Å and was thus substantially worse than for devitrified samples. In movie stacks of vitreous samples, frames 4–7 were consistently better than earlier or later frames.

**Figure 8:**
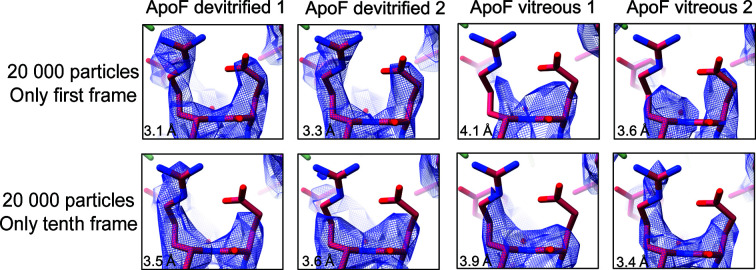
Density maps reconstructed from two data sets each of apoferritin in cubic ice (ApoF devitrified 1 and 2) and apoferritin in vitreous water (ApoF vitreous 1 and 2) with a fitted 1.5 Å resolution X-ray structure of horse apoferritin (PDB entry 2w0o). Rows 1 and 2 show reconstructions from 20 000 particles from the first or tenth movie frame. Density is drawn at the default contour level where 1% of the voxels are above the threshold.

Two representative arginine and glutamate side chains from the first-frame-only and tenth-frame-only maps are shown in Fig. 8 ▸. In the first-frame maps from devitrified samples both side chains are well resolved. In the tenth-frame reconstruction the glutamate side chain appears to be decarboxylated as a result of radiation damage, and in one map the arginine side chain is only partially resolved. By contrast, for vitrified samples the tenth-frame maps are better than the first-frame maps, because at that stage the beam-induced movement had settled down.

## 4. Discussion   

We report an approximately fourfold reduction of beam-induced specimen movement in the critical initial frames of a movie stack for cryo-EM samples in devitrified water compared with standard vitrified samples. In all three devitrified grids of two different protein samples examined in this study, the burst-like beam-induced movement in early frames at an accumulated fluence below ∼5 e Å^−2^ was substantially reduced.

Beam-induced specimen motion blurs the image and results in information loss, especially at high spatial frequencies. The high-resolution signal decays most rapidly in the first few frames that are most affected by motion. In *RELION* this is reflected in higher (*i.e.* less good) *B* factors (Scheres, 2014 ▸; Zivanov *et al.*, 2019 ▸). The relative *B* factors are then used in a frequency-dependent dose-weighting scheme, which downweights high spatial frequencies both in the blurred early frames and the radiation-damaged later frames. When beam-induced movement is minimized, the high-resolution signal in the early frames is at least partly preserved, as reflected in better relative *B* factors. Early frames which have suffered least from radiation damage then contribute more to the final map of devitrified specimens.

Many factors influence the quality of an electron cryo-micrograph, and therefore great care has to be taken when comparing micrographs from different grids acquired in separate microscope sessions. Different ice thicknesses, grid handling, electron-optical settings or different image-acquisition routines can all affect the quality of the resulting high-resolution density map. Even though we attempted to keep all these parameters constant, the quality of individual grids, grid squares and image stacks inevitably varies. This is demonstrated by the resolution achieved with horse apoferritin on seven different vitrified samples, which ranged from 2.52 to 2.83 Å with 51 200 particles (Fig. 4 ▸). Presumably, devitrified samples would vary as much when a similar number of grids are compared. The point of our present study is, however, to show that the first four frames (fluence of <5 e Å^−2^) from devitrified grids are consistently better than those from vitrified samples. This is clearly demonstrated by Figs. 7 ▸ and 8 ▸.

The behavior of vitreous water upon warming is difficult to control and devitrification on an EM grid can be erratic. Occasionally, grids did not devitrify even though they were kept on the grid heater for minutes, presumably owing to small differences in local surface temperature. Often, large contaminating ice crystals formed on the grid squares, most likely from atmospheric water, even though all experiments were conducted in the same way at constant, low humidity in an atmosphere of cold, dry nitrogen gas. On these grid squares water within the foil holes remained vitreous. Surprisingly, we never found contaminating ice and clean cubic ice on the same grid square. At present it is not clear why some grid squares do not contaminate and warm up sufficiently for the formation of cubic ice. It is most likely that this is due to local thermal gradients that vary from grid to grid. Another complication arises from the requirement that for high-resolution imaging areas of successful devitrification need to coincide with areas of suitable ice thickness. New approaches to the preparation of better cryo-EM specimens (Razinkov *et al.*, 2016 ▸; Ravelli *et al.*, 2020 ▸), which improve the reproducibility and homogeneity of ice thickness, would also increase the success rate for high-resolution imaging of devitrified samples.

Ice contamination remained problematic when the grid was warmed up in liquid ethane or in the vacuum of a microscope column. Attempts to devitrify grids in freshly prepared liquid ethane at a controlled temperature above the glass-transition temperature of water resulted in ice contamination that was more subtle than in a dry nitrogen atmosphere but was evident at low magnification. The contamination resembled ‘leopardskin ice’ (Fig. 2 ▸), which often forms as a result of water vapor deposited on the specimen in a poor microscope vacuum. It became visible when the specimen was heated to −130 to −120°C in liquid ethane (boiling point −89°C) and was dominant within 3–4 s at −115 to −110°C. Unexpectedly, the vitreous film within the foil holes did not devitrify under these conditions. Keeping the grids at −120 to −130°C under liquid ethane for 30–120 s resulted in ice contamination similar to that forming on the grid heater (Fig. 1 ▸), but the aqueous film did not devitrify. Control experiments indicated that ice contamination was not a result of grid transfer from liquid nitrogen to liquid ethane or to the grid heater, and under our conditions depended solely on the temperature and duration of the experiment.

In principle, it should be possible to avoid ice contamination when grids are devitrified in the high vacuum of an electron microscope, and this has worked in previous experiments (Cyrklaff & Kühlbrandt, 1994 ▸). However, under our present conditions, ice contamination was still a problem when we attempted to devitrify grids in a Gatan side-entry cryo-transfer holder in the column of our CM120 electron microscope, possibly because of poor column vacuum. It would be interesting to repeat these experiments in the column vacuum of a modern high-end electron cryo-microscope that allows control of the specimen temperature. Such instruments are not available at present.

To take full advantage of the improved stability of the cubic ice lattice, it would be desirable to devitrify specimens on all-gold grids (Russo & Passmore, 2014 ▸), which are considerably more stable than Quantifoil copper grids, and to image them in a liquid helium-cooled microscope at a temperature between 4 and 17 K. Cooling with liquid helium is known to protect 2D crystals of bacterio­rhodopsin against radiation damage by a factor of ∼2 in electron dose compared with liquid nitrogen (Stark *et al.*, 1996 ▸; Vinothkumar & Henderson, 2016 ▸). For single particles, this potential benefit of helium cooling is neutralized by a large, unexpected increase in beam-induced specimen movement (Pfeil-Gardiner *et al.*, 2019 ▸), but joint use of specimen devitrification and liquid-helium cooling might overcome this problem.

With the radiation-sensitive biological samples studied by cryo-EM, it is particularly important to extract as much information as possible from the precious early frames of a movie stack that are recorded before the sample has suffered extensive radiation damage. By transforming vitreous water to mechanically more stable cubic ice, stress trapped in the specimen during rapid vitrification is released prior to electron irradiation and this reduces beam-induced motion in early frames. The novel all-gold hexAuFoil specimen supports that reduce beam-induced motion to less than 1 Å r.m.s.d. appear to resolve this long-standing issue (Naydenova *et al.*, 2020 ▸). Nevertheless, controlled devitrification may become useful in situations where larger foil holes (more than 300 nm) or very thin ice layers (10 nm or less) are required, or with samples that cannot be put into holey foils at all.

## Supplementary Material

Supplementary Figures. DOI: 10.1107/S2052252520016243/pw5016sup1.pdf
