# Supplementary material for: Devitrification reduces beam-induced movement in cryo-EM
Source: IUCrJ. 2021 Mar 1;8(Pt 2):186–94. doi: 10.1107/S2052252520016243 (PMC7924229; doi:10.1107/S2052252520016243)
Supplement: Supplementary file 1 [file m-08-00186-sup1.pdf]

IUCrJ

Volume 8 (2021)

Supporting information for article:

Devitrification reduces beam-induced movement in cryo-EM

Jan Philip Wieferig, Deryck J. Mills and Werner Kühlbrandt

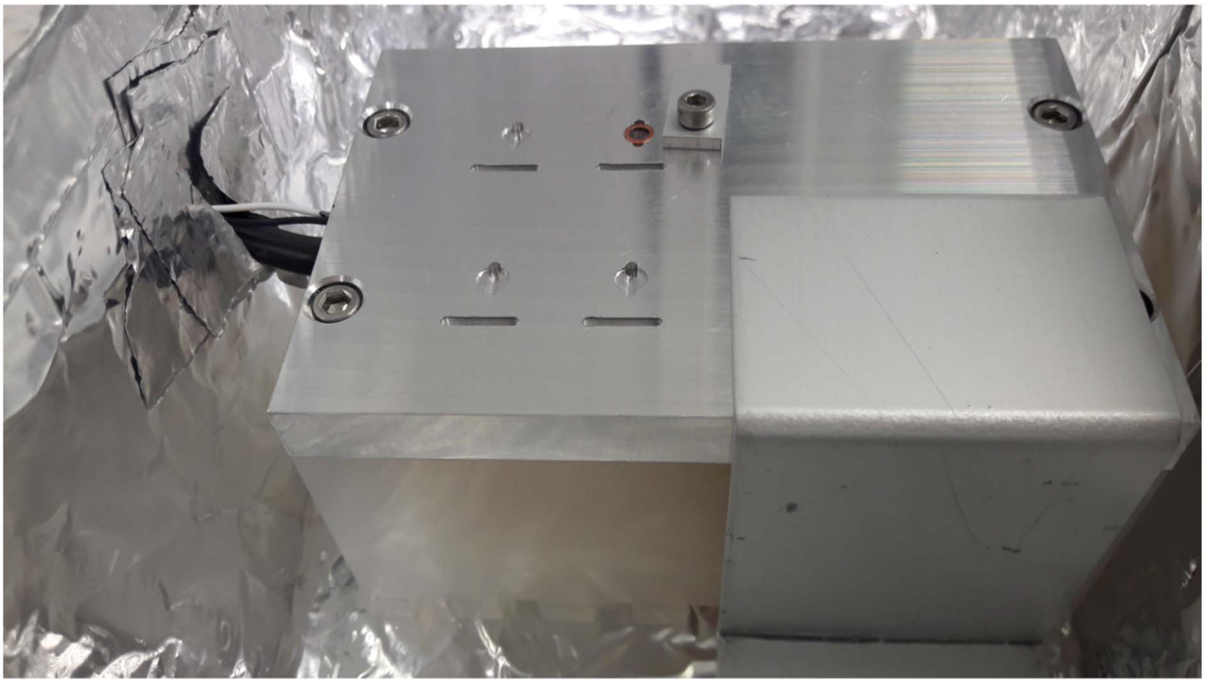

Figure S1 Home-built grid heater for controlled devitrification. The grid heater consists of an aluminium plate on top of a plastic block containing the resistance element for heating the metal plate. The grid heater is placed into a styrofoam box lined with aluminium foil to minimize nitrogen bubbling. During devitrification, the liquid nitrogen level is kept ~1 cm below the top of the plastic block, and the styrofoam box is covered with an acrylic glass pane. At a heater power of 7 W, heat transfer from the plastic base is sufficient to heat the metal plate to - 120°C. The temperature is measured with a pt100-Resistance Temperature Detector inserted centrally into the metal plate. Grids are accommodated in wells milled into the plate and are held in place by a rotating metal lid secured with a screw. Heat transfer is improved by a Z-shaped metal sheet that is partly immersed in liquid nitrogen and partly covers the top plate.

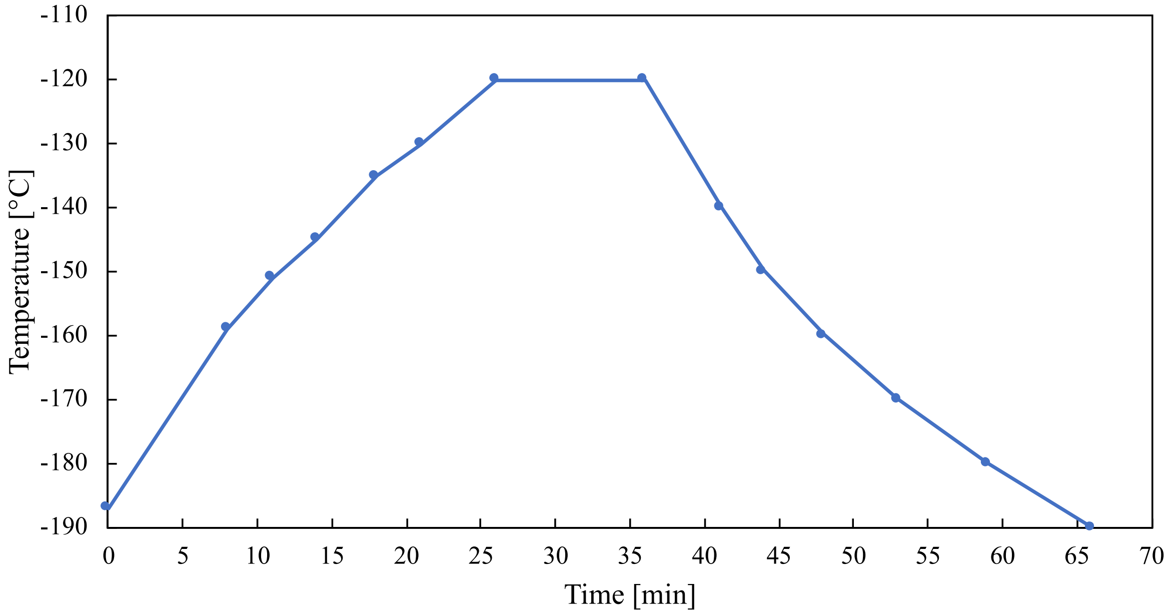

Figure S2 Temperature course of a devitrification cycle, which resulted in the successful devitrification of the alcohol oxidase specimen in Figure 5A and 6A. The grid was placed in the well of the grid heater at liquid nitrogen temperature and covered with the rotating lid. The temperature of the top plate was raised and kept at - 120°C for 10 min, after which the power supply was shut off and the z-shaped metal sheet was positioned as in **Fig. S1** to improve heat transfer between the metal plate and liquid nitrogen. The grid was returned to the liquid nitrogen storage when the top plate had reached - 190°C. In other successful devitrification experiments, the grid was placed onto the top plate at a temperature of - 100 to - 110°C for a few seconds.

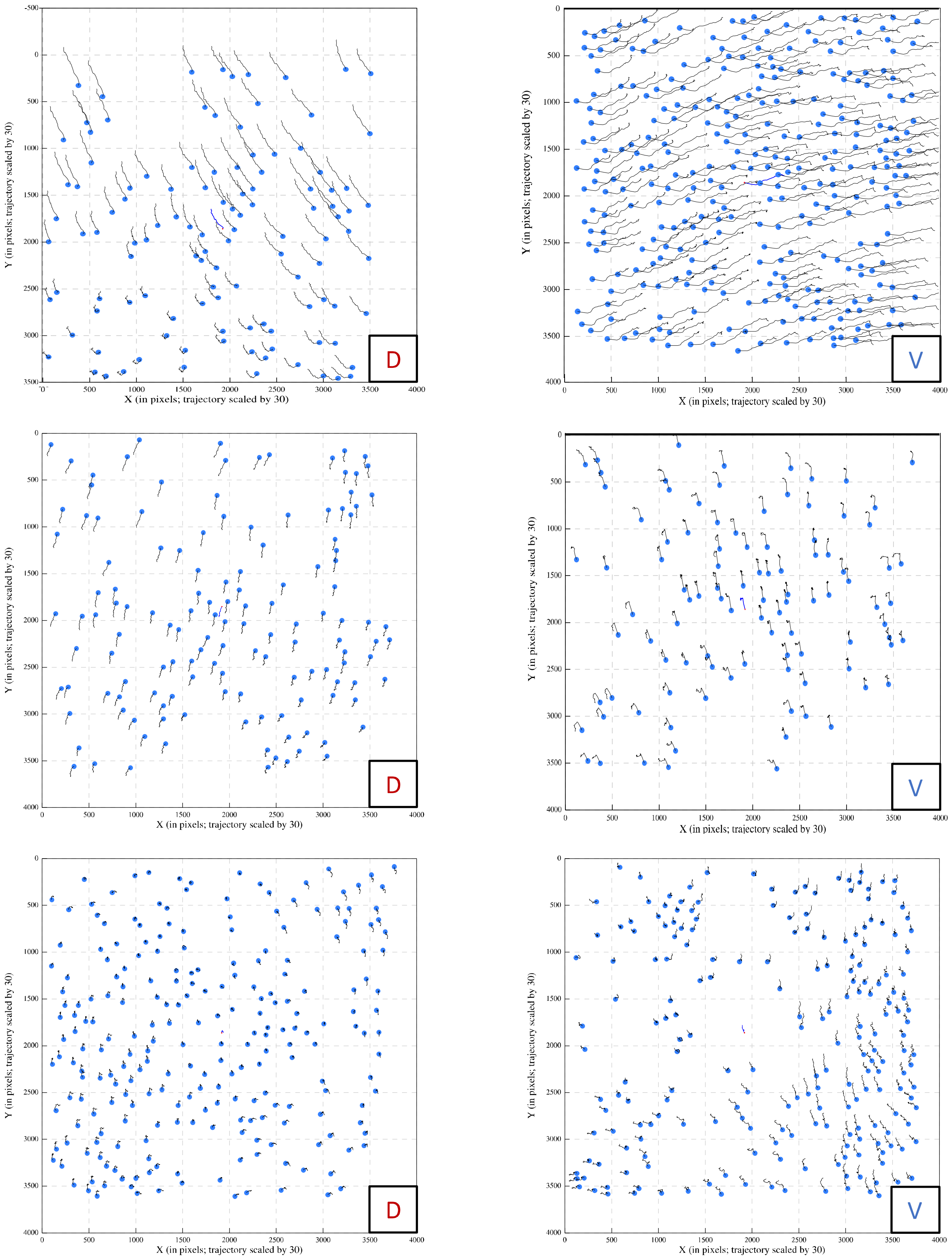
Y (in pixels; trajectory scaled by 30)
X (in pixels; trajectory scaled by 30)
D
Y (in pixels; trajectory scaled by 30)
X (in pixels; trajectory scaled by 30)
V
Y (in pixels; trajectory scaled by 30)
X (in pixels; trajectory scaled by 30)
D
Y (in pixels; trajectory scaled by 30)
X (in pixels; trajectory scaled by 30)
V
Y (in pixels; trajectory scaled by 30)
X (in pixels; trajectory scaled by 30)
D
Y (in pixels; trajectory scaled by 30)
X (in pixels; trajectory scaled by 30)
V

A

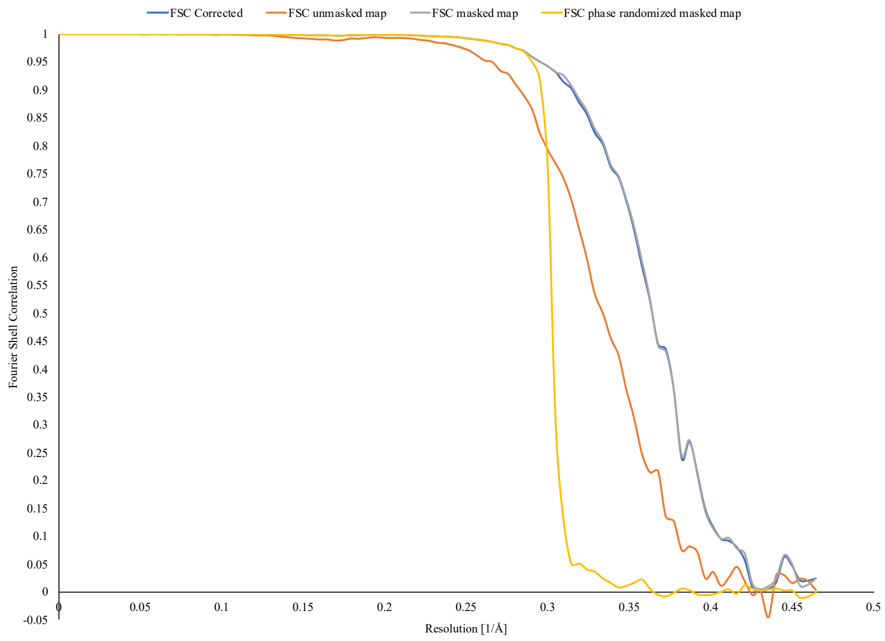

B

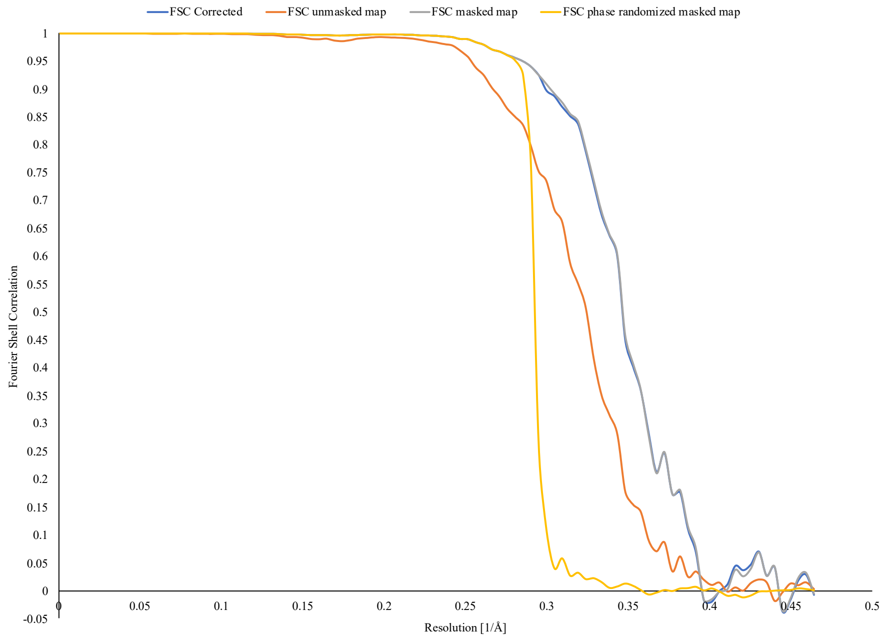

Figure S4 Fourier shell correlations of the 2.5 Å map from dataset ApoF vitreous 2 (A) and the 2.6 Å map from dataset ApoF devitrified 2 (B) which were reconstructed from 52,100 particles in vitreous buffer and cubic ice, respectively.
